# The Pivotal Role of 5-Lipoxygenase-Derived LTB_4_ in Controlling Pulmonary Paracoccidioidomycosis

**DOI:** 10.1371/journal.pntd.0002390

**Published:** 2013-08-22

**Authors:** Patrícia Campi Santos, Daniel Assis Santos, Lucas Secchim Ribeiro, Caio Tavares Fagundes, Talles Prosperi de Paula, Thiago Vinícius Avila, Ludmila de Matos Baltazar, Mila Moreira Madeira, Rosana de Carvalho Cruz, Ana Carolina Fialho Dias, Fabiana Simão Machado, Mauro Martins Teixeira, Patrícia Silva Cisalpino, Danielle G. Souza

**Affiliations:** 1 Laboratory of Microorganism-Host Interaction, Department of Microbiology, Institute of Biological Sciences, Federal University of Minas Gerais, Minas Gerais, Brazil; 2 Laboratory of Immunopharmacology/Department of Biochemistry and Immunology, Institute of Biological Sciences, Federal University of Minas Gerais, Minas Gerais, Brazil; 3 Inflammation Research Group, School of Biochemistry and Immunology, Trinity Biomedical Sciences Institute, Trinity College Dublin, Dublin, Ireland; University of California San Diego School of Medicine, United States of America

## Abstract

Leukotrienes (LTs) produced from arachidonic acid by the action of 5-lipoxygenase (5-LO) are classical mediators of inflammatory responses. However, studies published in the literature regarding these mediators are contradictory and it remains uncertain whether these lipid mediators play a role in host defense against the fungal pathogen *Paracoccidioides brasiliensis*. To determine the involvement of LTs in the host response to pulmonary infection, wild-type and LT-deficient mice by targeted disruption of the 5-lipoxygenase gene (knockout mice) were studied following intratracheal challenge with *P. brasiliensis* yeasts. The results showed that infection is uniformly fatal in 5-LO-deficient mice and the mechanisms that account for this phenotype are an exacerbated lung injury and higher fungal pulmonary burden. Genetic ablation or pharmacological inhibition of LTs resulted in lower phagocytosis and fungicidal activity of macrophages *in vitro*, suggesting that deficiency in fungal clearance seems to be secondary to the absence of activation in 5-LO^−/−^ macrophages. Exogenous LTB_4_ restored phagocytosis and fungicidal activity of 5-LO^−/−^ macrophages. Moreover, *P. brasiliensis* killing promoted by LTB_4_ was dependent on nitric oxide (NO) production by macrophages. Taken together, these results reveal a fundamental role for 5-LO-derived LTB_4_ in the protective response to *P. brasiliensis* infection and identify relevant mechanisms for the control of fungal infection during the early stages of the host immune response.

## Author Summary

Paracoccidioidomycosis is a deep mycosis that is endemic in Latin America, mostly affecting rural workers of Argentina, Colombia, Venezuela and Brazil. *Paracoccidioides brasiliensis* infection is acquired upon the inhalation of airborne propagules derived from the saprophytic mycelium form of the fungus. Once in the lungs, *P. brasiliensis* converts to its parasitic yeast form and interacts with alveolar macrophages. This phagocytic cell is specialized in secreting cytokines, lipid mediators, and microbicidal molecules that control the fungal growth. Among the lipid mediators generated by macrophages, 5-LO-derived leukotrienes play an important role in lung innate immunity and are known to be important for neutrophil recruitment and activation. Here, we evaluated whether leukotrienes are involved in the immune response in a murine model of *P. brasiliensis* infection. 5-LO-deficient mice exhibited high mortality, severe lung pathogenesis and increased fungal burden in the lungs, associated with less activation of macrophages, resulting in lower phagocytosis and *P. brasiliensis* killing. These results reveal for the first time that endogenous production of leukotrienes, especially LTB_4_, is important for assembling a protective immune response of the host against *P. brasiliensis*.

## Introduction

Paracoccidioidomycosis (PCM) is a systemic granulomatous disease caused by the dimorphic fungi *Paracoccidioides brasiliensis* and *Paracoccidioides lutzii*. PCM is the most prevalent deep mycosis in Latin America [1], [2] and the most important cause of death among systemic mycoses in immunocompetent individuals in Brazil [3]. PCM is considered a neglected infectious disease, as the development of new drugs for the treatment of this mycosis has received little attention [4], [5]. Epidemiological and experimental data suggest that humans probably become infected by inhalation of airborne conidia (the infective form) that reach the lung alveoli, where they transform into yeast cells, the parasitic tissue form [6]. Therefore, it is clear that this route of infection is more relevant experimentally. Clinical forms of the disease range from asymptomatic pulmonary infection to systemic generalized disease [7]. In the asymptomatic form of PCM, T-helper type 1 (Th1) specific immune response occurs, while Th2 immunity is associated with severe disease [8]–[10]. Cellular immune response plays a major role in the host defense against *P. brasiliensis* and macrophages are the most important effector cells that kill the fungus by oxidative mechanisms and cytokine production [11], [12]. Experimental studies and data from patients with PCM demonstrate that resistance to *P. brasiliensis* infection is dependent on the activity of T helper cells and mediated by IFN-γ, TNF-α and macrophages/monocytes. The synergistic effect between these cytokines is essential for host resistance and effective fungicidal activity against *P. brasiliensis*. During the course of infection, CD4^+^ T lymphocytes synthesize cytokines, such as IFN-γ, TNF-α and IL-12, which provide protection to the host, and prevents fungus spread [13], [14].

In addition to cytokines, lipid mediators such as leukotrienes (LTs) are another class of molecules involved in host defense [15]. LTs are derived from the metabolism of a cell-membrane fatty acid, arachidonic acid (AA), through activation of 5-lipoxygenase (5-LO) enzyme, in concert with its auxiliary protein, 5-LO-activating protein (FLAP). 5-LO catalyzes oxidation of AA to the intermediate 5-hydroperoxyeicosatetraenoic acid (5-HPETE), which is either enzymatically reduced by 5-LO to the unstable epoxide leukotriene A_4_ (LTA_4_) or, alternatively, is reduced to 5-hydroxyeicosatetraenoic acid (5-HETE). LTA_4_ can be hydrolyzed to form leukotriene B_4_ (LTB_4_) or can be conjugated with glutathione to form the cysteinyl leukotrienes (cysLTs), LTC_4_, LTD_4_ and LTE_4_ [16]. LTB_4_ is a potent effector of leukocyte chemotaxis and activation, while the cysLTs increase vascular permeability and smooth muscle tone. The activity of leukotrienes is signaled through two sets of G-protein coupled receptors, BLT_1_/BLT_2_ for LTB_4_ and CysLT_1_/CysLT_2_ for cys-LTs [17].

LTs are produced at sites of infection and *in vitro* and *in vivo* models have revealed a powerful immunoregulatory role for LTB_4_ [18]–[27]. However, two recent studies published reported conflicting participation of 5-LO in *P. brasiliensis* infection. Although 5-LO enzymatic activity was shown to enhance susceptibility during experimental *P. brasiliensis* infection [28], Balderramas and colleagues demonstrated that leukotriene production was associated with a protective response during the early stages of *P. brasiliensis* infection in the lungs. LTB_4_ was shown to induce influx and activation of phagocytes [29]. Therefore, the relevance of 5-LO and its metabolic products during infection by *P. brasiliensis* remains undefined. In the present study, we evaluated the role of LTs in host defense against the pathogenic fungus *P. brasiliensis* inoculated in the lungs. As an experimental approach, we compared survival and components of the host response in wild-type and 5-LO-deficient mice, employing a model of intratracheal challenge with *P. brasiliensis* yeasts, which mimics the infection route of the fungus in human host. We sought to investigate the importance of LTs in phagocytosis and fungicidal activity through genetic and pharmacological tools and *in vitro* challenge of macrophages with *P. brasiliensis* yeasts. 5-LO deficiency was associated with macrophage hyporesponsiveness during *P. brasiliensis* infection, resulting in lower phagocytosis and fungal clearance, leading to increased fungal burden in the lungs and mortality of mice. The results reveal that endogenous 5-LO metabolites, especially LTB_4_, play a fundamental role in the protective host response to *P. brasiliensis* infection.

## Methods

### Animals

Male Sv129 (Wild-type - WT) and 5-LO-deficient mice (5-LO^−/−^) (6 to 8 weeks old) were used in all experiments. WT mice were obtained from the Animal Care Facilities of Federal University of Minas Gerais, Minas Gerais, Brazil and 5-LO^−/−^ mice were purchased at The Jackson Laboratory, Bar Harbor, Maine, USA [30]. Mice were kept under controlled environmental conditions (temperatures of 24°C and 12 h light/dark cycle) and supplied with sterile food and water in clean bottles, as well as clean bedding.

### Ethics statement

Animal experiments were performed in strict accordance with the Brazilian Federal Law 11,794 establishing procedures for the scientific use of animals and approved by the Animal Care and Use Committee of the Federal University of Minas Gerais (protocol no. 194/09).

### Fungal culture and quantification

The Pb18 strain, a highly virulent isolate of *P. brasiliensis*, is part of the strains collection of *P. brasiliensis* in our laboratory. Pb18 yeast cells were maintained by weekly subcultivation on 2% glucose, 1% peptone, 0.5% yeast extract medium culture (YPD medium). The yeast cells were washed in sterile phosphate-buffered saline (PBS) (pH 7.2) and homogenized. Cells were adjusted to 1×10^6^/ml (for *in vivo* infection) and 6×10^4^/ml (for *in vitro* infection) based on hemocytometer counts. Cell viability was determined with Janus Green B vital dye (Merck) and was always higher than 90%.

### Reagents

Zileuton, CP105,696 and montelukast were provided by Abbott Pharmaceuticals, Pfizer and Merck, respectively. LTB_4_ and recombinant interferon-gamma (IFN-γ) was purchased from Cayman Chemical and BD Pharmigen, respectively. Nω-Nitro-L-arginine methyl ester hydrochloride (L-NAME), a nitric oxide synthase inhibitor and 4′-Hydroxy-3′-methoxyacetophenone (apocynin), a NADPH oxidase inhibitors were purchased by Sigma Aldrich.

### Treatment with zileuton, an inhibitor of 5-lipoxygenase enzyme

The compound was diluted in 100 µl of absolute ethanol plus carboxymethylcellulose (Synth) at 0.5%, in order to get the treatment solution. WT mice were treated daily with 30 mg/Kg/0.2 ml by gavage, the first and last dose administered 1 hour before infection with *P. brasiliensis* yeasts and before euthanasia of animals, respectively. As a control, mice injected with PBS or infected with *P. brasiliensis* were treated daily with the same volume of vehicle solution by the same route.

### Experimental infection

Mice were anesthetized by intraperitoneal (i.p.) injection of a solution containing ketamine hydrochloride (100 mg) and xylazine (20 mg) (Syntec, São Paulo, Brazil). When deep anesthesia was obtained, an anterior midline incision was made for trachea exposition. WT and 5-LO^−/−^ mice were infected with 10^6^ *P. brasiliensis* yeast cells, in a volume of 30 µl by surgical intratracheal (i.t.) inoculation, which allowed dispensing of the fungal cells directly into the lungs. Uninfected mice (control) received PBS only. The skin was then sutured, and mice were allowed to recover on a heated plate.

### Lung lavage and cell differential count

At different periods after infection, animals were euthanized. The chest cavity of each animal was carefully opened, and the trachea was exposed and catheterized. Lungs were washed after the canulation of trachea with polyethylene tubing, which was attached on a tuberculin syringe. The catheter was tied in place, and sterile PBS was infused in 1 ml aliquots three times. Bronchoalveolar lavage fluid (BAL) was recovered and total cell counts were immediately performed in a Neubauer chamber. Differential counts for leukocyte subsets were obtained by using cytospin preparations.

### Immunolabeling and Fluorescence-Activated Cell Sorting Analysis

Bronchoalveolar cells were stained for extracellular molecular expression patterns using monoclonal antibodies against mouse F4/80, CD11c and MHC-II conjugated to fluorophores, and isotype controls (all from BD Pharmingen). Data were acquired on a FACSCanto II (Becton Dickinson) and analyzed by FlowJo 7.5.3 software (TreeStar Inc., Ashland, OR). Limits for the quadrant markers were always set on the basis of negative populations and isotype controls. Results are presented as mean fluorescence intensity (MFI) as indicated.

### Fungal burden assessment

The number of viable yeasts in BAL and lungs of infected mice were determined by colony-forming units (CFU) counts. After periods of infection, mice were euthanized, and their lungs were removed, weighed, and homogenized in 1 ml sterile PBS with a tissue grinder. Aliquots (100 µl) of each homogenate and BAL were placed on brain-heart infusion (BHI) agar medium (Difco Laboratories), containing 4% (vol/vol) fetal bovine serum (Cultilab, São Paulo, Brazil), 40 µg/ml gentamicin (Sigma-Aldrich) and 5% *P. brasiliensis* B339 broth yeast culture filtrate from 2-week-old cultures (supplemented BHI), the latter constituting the source of a growth-promoting factor [31]. The plates, in duplicate, were incubated at 37°C and colonies were counted daily until no increase in counts was observed. The numbers of viable *P. brasiliensis* per gram of tissue or ml of BAL were expressed as the mean ± the standard error of the mean (SEM).

### Myeloperoxidase (MPO) concentrations

The extent of neutrophil accumulation in the lungs was measured by assaying myeloperoxidase activity. Briefly, a portion of the lungs were removed and snap frozen in liquid nitrogen. On thawing and processing, the tissue was assayed for myeloperoxidase activity by measuring the change in optical density (O.D.) at 450 nm using tetramethylbenzidine (Sigma-Aldrich). Results were expressed as the neutrophil index that denotes activity of myeloperoxidase related with casein-elicited murine peritoneal neutrophils processed in the same way.

### Measurement of cytokines

The concentrations of murine TNF-α, IFN-γ, IL-1β, IL-6, IL-10 and keratinocyte-derived chemokine (CXCL-1/KC) and macrophage inflammatory protein-2-gamma (CXCL-2/MIP-2) was measured in BAL and lungs samples using commercially available antibodies and according to the procedures supplied by the manufacturer (R&D Systems, Minneapolis, MN). One hundred milligrams of lung were homogenized in 1 ml of PBS containing anti-proteases (0.1 mmol/L phenylmethil sulfonyl fluoride, 0.1 mmol/L benzethonium chloride, 10 mmol/L ethylenediaminetetraacetic acid, and 20 KI aprotinin A) and 0.05% Tween 20. The samples were then centrifuged for 10 minutes at 13000× g and the supernatant stored at −20°C until enzyme-linked immunosorbent assay (ELISA) was performed.

### Isolation and cultivation of murine macrophages

Peritoneal macrophages were elicited by i.p. injection of 3% sterile thioglycollate medium (2 mL). After 3 days, mice were euthanized and macrophages were harvested by peritoneal lavage with PBS. Alveolar macrophages were isolated from the bronchoalveolar fluid of animals after the lung lavage protocol described above. Cells (2×10^5^ cells/well in 24-well plates) were either isolated by adherence (4 h at 37°C under 5% CO_2_) to plastic-bottom tissue culture plates (for fungicidal assays) or plated onto 13-mm-diameter round glass coverlips (for phagocytosis assays). Macrophages were washed to remove nonadherent cells and were cultivated overnight with fresh complete medium (RPMI-1640 tissue culture medium [Cultilab, Brazil] supplemented with 2 mM L-glutamine [Sigma-Aldrich], 10% fetal bovine serum, 20 mM HEPES [Sigma-Aldrich] and 40 µg/ml gentamicin [Sigma-Aldrich]) in the presence or absence of recombinant IFN-γ (100 U/ml).

### Infection of murine macrophages cultures with *P. brasiliensis*

For phagocytosis assays, *P. brasiliensis* yeasts were added at a ratio of 1∶5 macrophages to fungal cells. The cultures were incubated for 24 h at 37°C under 5% CO_2_ to allow adhesion and ingestion of fungi. Cells were washed twice with PBS to remove any noningested or nonadherent yeasts, fixed with methanol and stained with Giemsa (Sigma Aldrich). Internalized yeasts were counted by light microscopy and the phagocytic index (PI) was defined by the equation PI = P X F, where P is the percentage of macrophages with internalized yeast and F is the average number of yeast cells per macrophage. At least three independent experiments were carried out in triplicate for each condition [32]. For fungicidal assays, IFN-γ-primed and unprimed macrophages were infected with *P. brasiliensis* yeasts as described above. After the infection, the supernatants were removed and stored at −20°C and further analyzed for the presence of nitric oxide (NO). The wells were washed with water to lyse macrophages and one hundred microliters of cell homogenates were placed on BHI supplemented agar medium and assayed for the presence of viable yeasts after 7–10 days of incubation at 37°C. Fungicidal assay was normalized according differences in initial ingestion and presented as *P. brasiliensis* survival. In some experiments, cells were pretreated for 30 minutes before infection with the 5-LO enzyme inhibitor zileuton (10 µM), LTB_4_ receptor antagonist CP-105,696 (1 µM), cysteinyl-LT receptor antagonist montelukast (10 µM), LTB_4_ (100 nM), iNOS inhibitor L-NAME (1 mM) or NADPH oxidase inhibitor apocynin (1 mM). Compounds requiring reconstitution were dissolved in either PBS or dimethyl sulfoxide (DMSO). Required dilutions of all compounds were prepared immediately before use, and equivalent quantities of vehicle were added to the appropriate controls.

### Measurement of LTB_4_

For LTB_4_ measurement, lungs were removed and tissues homogenized, centrifuged at 10000× g for 10 minutes and stored at −80°C until assayed. The supernatant obtained from the phagocytosis assay was also used for LTB_4_ analysis. Quantification of LTB_4_ in the samples was performed by specific enzyme immunoassay (Leukotriene B_4_ EIA Kit, Cayman Chemical, Ann Arbor, Mich.) following the manufacturer's instructions. The sensitivity for LTB_4_ was 4 pg/ml.

### Macrophage nitric oxide production

Nitric oxide (NO) was quantified by the accumulation of nitrite (NO_2_^−^) in the supernatants of macrophage cultures using Griess assay. Briefly, 100 µl of the supernatants collected were mixed with equal volume of Griess reagent (1% sulfanilamide [Sigma-Aldrich] diluted in 5% of phosphoric acid [H_3_PO_4_], and 0.1% Naphthylethylenediamine [Sigma-Aldrich]) in 96-well plates and absorbance at 540 nm was determined. Conversion of absorbance to micromolar concentrations of nitrite was obtained using a standard curve of a known concentration of NaNO_2_ diluted in water. All measurements were performed in duplicate and expressed as micromolar concentrations of nitrite.

### Intracellular oxidative burst assay using flowcytometric analysis

The intracellular production of reactive oxygen species (ROS) was assayed by using the substrate dihydrorhodamine 123 (DHR-123, Invitrogen, Eugene, USA) that diffuses into the cells and is oxidized by ROS to the fluorescent Rhodamine 123 [33]. Macrophages were cultivated according to phagocytosis protocol in 96-well plates and after 24 h of infection, the cells were incubated with 5 µM DHR-123 for 30 min at 37°C in the dark. The fluorescence was measured in a flowcytometer (Synergy 2) at an excitation wavelength of 480 nm and at emission wavelength of 530 nm.

### Histological analysis

Lungs were removed after infection and tissues were fixed in 10% formalin and embedded in paraffin blocks. Tissue sections (5 µm thick) were stained with hematoxylin and eosin (H&E) for histopathological evaluation. Sections were captured with a digital camera (DEI-470; Optronics, Goleta, CA) connected to a microscope (IX70; Olympus, Center Valley, PA). Inflammation was scored as follows: 0: no inflammation; 1, perivascular cuff inflammatory cells; 2, mild inflammation, extending throughout <25% of the lung; 3, moderate inflammatory covering 25–50% of the lung; 4, severe inflammation over one-half of the lung.

### Statistical analysis

The results were analyzed by GraphPad Prism 4 (GraphPad Inc., San Diego, CA, USA). Data were presented as mean ± standard error of the mean (SEM) and were compared using one-way ANOVA test with Newman-Keuls multiple comparisons post-test. P values<0.05 were considered significant.

## Results

### 5-LO deficiency decreased animal survival and pulmonary fungal clearance

**Figure 1:**
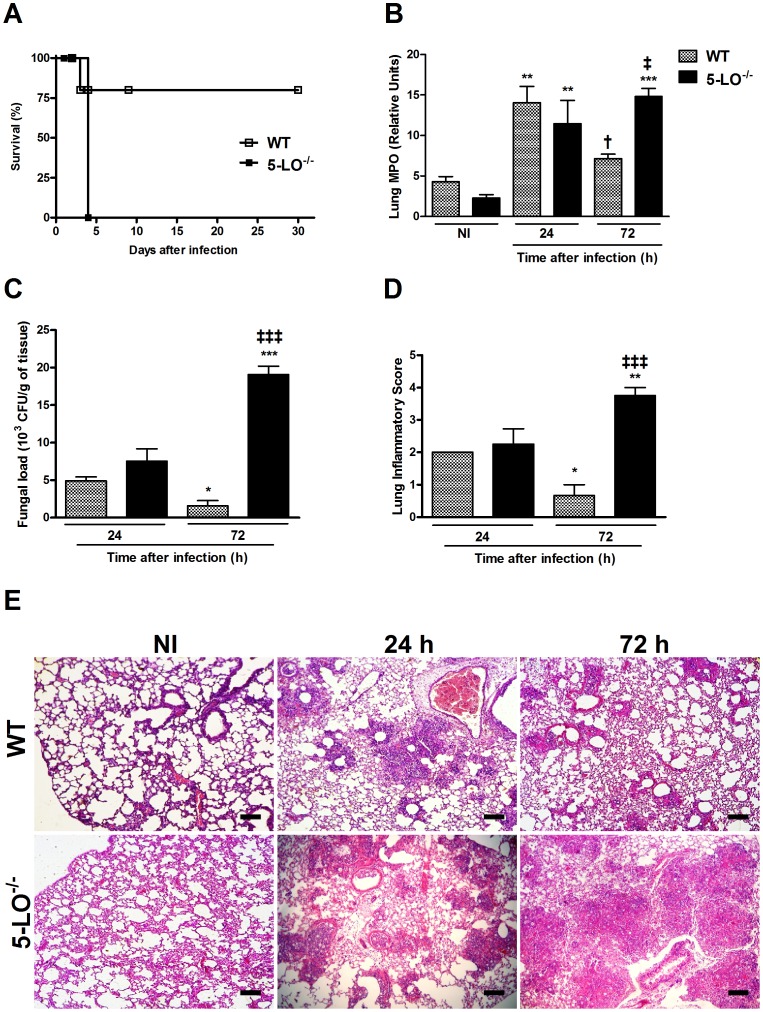
Role of 5-LO in survival, myeloperoxidase activity, fungal load and histopathological alterations after *P. brasiliensis* infection. Groups of WT and 5-LO^−/−^ mice were infected with 10^6^ yeasts. (A) Survival rate was assessed daily until the 30^th^ day of infection. (B) Infiltration of neutrophils in the lungs of mice was determined by MPO activity and expressed in relative units. (C) Pulmonary fungal load in WT and 5-LO^−/−^ mice. Viable yeasts were evaluated by CFU counting in lung homogenates obtained 24 and 72 h after infection. (D) Inflammatory score of lung sections evaluated 24 and 72 h after infection in WT and 5-LO^−/−^ mice. (E) Representative photomicrographs of H&E-stained lung sections from WT (upper line) and 5-LO^−/−^ (bottom line) mice 24 and 72 h after *P. brasiliensis* infection (bars = 100 µm, original magnification, ×10). Control, uninfected mice (NI) received only sterile PBS. Results were expressed as the mean ± SEM and are representative of two independent experiments (n = 5). Symbol * represents significant difference (*p<0.05, **p<0.01, ***p<0.001) when compared to uninfected control or infected mice counterparts at 24 h after infection. Symbol ^†^ represents significant difference (^†^p<0.05) to infected mice counterparts in evaluated periods. Symbol ^‡^ represents significant difference (^‡^p<0.05, ^‡‡‡^p<0.001) between WT and 5-LO^−/−^ mice in the respective period evaluated.

Approximately 75% of WT mice infected with *P. brasiliensis* survived until day 30 after infection, the end of the observation period, while 100% of 5-LO^−/−^ mice died within four days after infection (Figure 1A). Infection with *P. brasiliensis* yeasts resulted in increased myeloperoxidase (MPO) activity in WT and 5-LO^−/−^ mice 24 h after intratracheal inoculation when compared with uninfected mice. Neutrophil accumulation peaked after 24 h in the lungs of WT mice and was significantly reduced after 72 h of *P. brasiliensis* infection (Figure 1B). 5-LO^−/−^ mice had a similar increase of MPO activity 24 h after infection but, unlike WT mice, there was higher MPO activity 72 h after infection.

In order to evaluate whether the increased mortality of 5-LO^−/−^ mice was due to fungal burden, viable yeast cells from bronchoalveolar lavage fluid (BAL) and lungs obtained from WT and 5-LO^−/−^ mice infected with *P. brasiliensis* yeasts were quantified by CFU counts (Figure 1C). At 24 h after infection, viable yeasts were recovered from WT lungs, followed by a significant decrease 72 h after infection. Similar amount of fungal yeasts was recovered after 24 h from lungs of 5-LO^−/−^ mice, but fungal load increased significantly and was about twelve times higher in 5-LO^−/−^ than WT mice 72 h after infection (Figure 1C). The same pattern was observed in BAL of WT and 5-LO^−/−^ mice (data not shown).

### 5-LO deficiency resulted in exacerbated lung inflammation in response to *P. brasiliensis* infection

**Figure 2:**
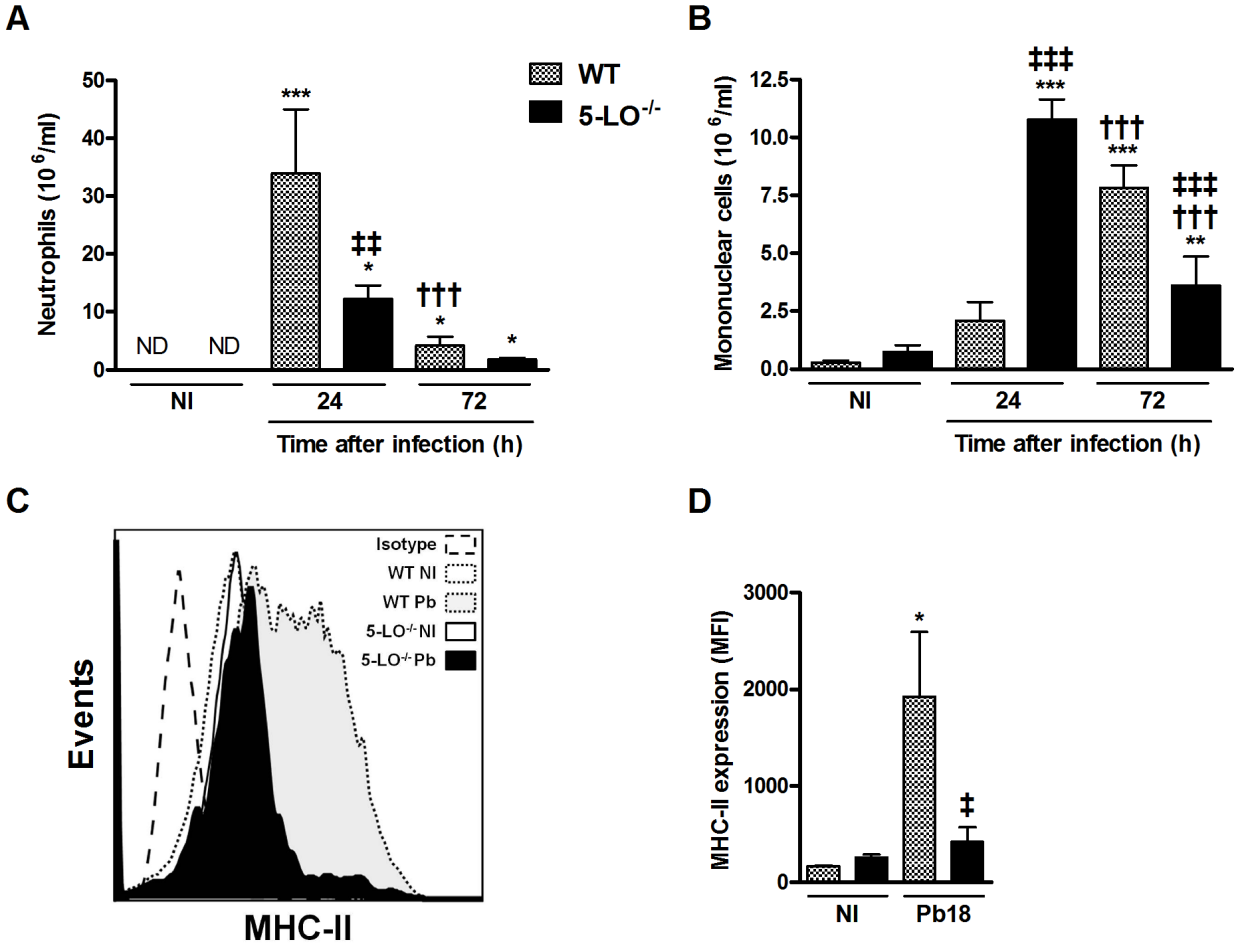
Effect of 5-LO on leukocyte recruitment and activation profile of bronchoalveolar cells. (A–B) BAL cells were obtained from mice 24 and 72 h after infection. Neutrophils and mononuclear cells were identified and counted after staining. (C) Flow cytometry analyses of bronchoalveolar leukocytes (F4/80^+^CD11c^+^ cells) from uninfected (NI), WT and 5-LO^−/−^ mice 24 h after infection with *P. brasiliensis*. Histograms were generated showing surface expression of MHC-II. (D) Activated F4/80^+^CD11c^+^ lymphocytes (macrophages) expression of MHC-II was also evaluated and expressed as MFI. Results were expressed as mean ± SEM and are representative of independent experiments (n = 5). Control, uninfected mice (NI) received only sterile PBS. Symbol * represents significant difference (*p<0.05, **p<0.01, ***p<0.001) when compared to uninfected control or infected mice counterparts at 24 h after infection. Symbol ^†^ represents significant difference (^†††^p<0.001) to infected mice counterparts in evaluated periods. Symbol ^‡^ represents significant difference (^‡^p<0.05, ^‡‡^p<0.01, ^‡‡‡^p<0.001) between WT and 5-LO^−/−^ mice in the respective periods evaluated. MFI, mean fluorescence intensity. ND, not detected.

Next, we scored the inflammation in the lungs of infected mice. Semi-quantification of lung inflammation concurred with the overall histopathological findings (Figure 1D–E). As shown in Figure 2E, lungs of uninfected WT and 5-LO^−/−^ mice were similar. At 24 h after infection, there was similar tissue inflammation in WT and 5-LO^−/−^ mice (Figure 1E). At 72 h after infection, lungs from WT mice showed dramatically reduced inflammatory infiltration and alveolar spaces were usually free of inflammation. In contrast, in 5-LO-deficient mice infected with *P. brasiliensis*, the extent of the inflammatory response was substantially greater with severe, widespread inflammation associated with a decrease in alveolar space and loss of lung architecture (Figure 1E).

### 5-LO deficiency impaired inflammatory cell recruitment and macrophage activation

Intratracheal inoculation of *P. brasiliensis* in WT mice induced significant neutrophil recruitment into the bronchoalveolar space 24 h after infection (Figure 2A). The number of neutrophils decreased in BAL but was still greater compared to uninfected mice 72 h after infection. In 5-LO^−/−^ mice, neutrophils counts was lower than WT mice 24 and 72 h after infection (Figure 2A). Considering the mononuclear cells influx, WT mice showed increased cell recruitment between 24 and 72 h after infection (Figure 2B). In 5-LO^−/−^ mice, mononuclear cells counts were higher at 24 h but greatly reduced 72 h after infection (Figure 2B).

After, we measured the activation of bronchoalveolar lavage cells of WT and 5-LO^−/−^ mice 24 h after infection, a time point when occurs the peak of the inflammatory events in WT mice. The activation of F4/80^+^CD11c^+^ alveolar macrophages was evaluated 24 h after infection by measuring surface expression of class II major histocompatibility (MHC-II). After *P. brasiliensis* infection, there was a significant increase of MHC-II expression on WT macrophages (Figure 2C and 2D). In contrast, there was no difference in the regulation of MHC-II expression in 5-LO^−/−^ macrophages after infection compared to uninfected cells (Figure 2C and 2D).

### 5-LO deficiency changed cytokines production in BAL and lungs during *P. brasiliensis* infection

**Figure 3:**
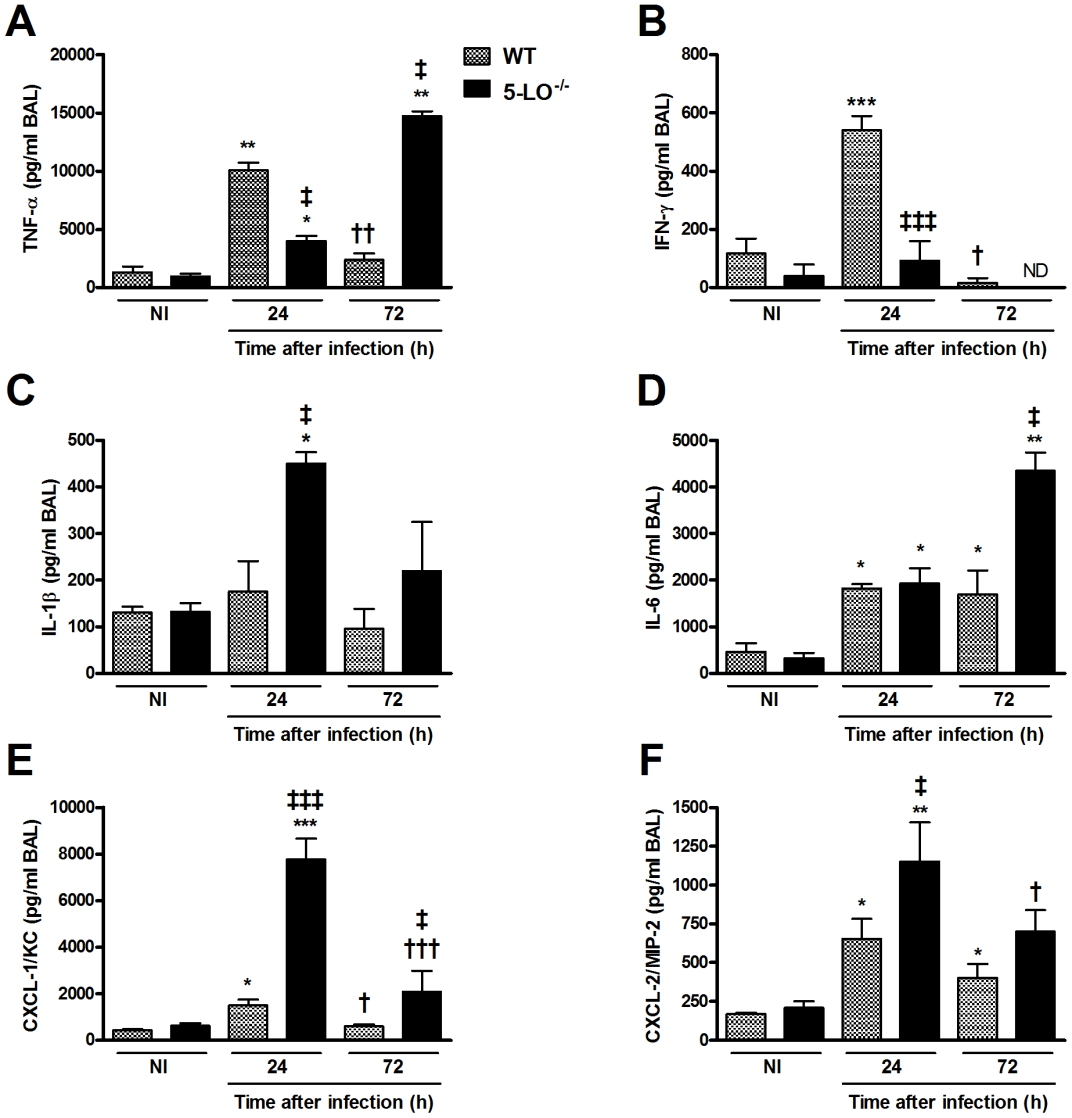
Cytokine levels in BAL after *P. brasiliensis* infection. WT and 5-LO^−/−^ mice were infected with 10^6^ *P. brasiliensis* yeasts. Cytokine levels for TNF-α (A), IFN-γ (B), IL-1β (C), IL-6 (D), CXCL-1/KC (E) and CXCL-2/MIP-2 (F) was determined by ELISA in BAL after 24 and 72 h of infection. The data were expressed as mean ± SEM (n = 5). Symbol * represents significant difference (*p<0.05, **p<0.01, ***p<0.001) between infected mice compared to respective control group. Symbol ^†^ represents significant difference (^†^p<0.05, ^††^p<0.01, ^†††^p<0.001) to infected mice counterparts in evaluated periods. Symbol ^‡^ represents significant difference (^‡^p<0.05, ^‡‡‡^p<0.001) between WT and 5-LO^−/−^ mice in the respective periods evaluated. ND, not detected.

Then, we determined whether 5-LO^−/−^ mice presented altered levels of cytokines previously shown to be involved in inflammatory and immune responses to *P. brasiliensis* (Figure 3 and Table 1). In WT mice, levels of TNF-α, IFN-γ and CXCL-1 peaked 24 h after infection and decreased to virtually background levels after 72 h in BAL (Figure 3A, B and E). Regarding the cytokine IL-1β, there was no difference in the concentration of this cytokine in WT-infected mice at the different times evaluated compared to control animals (Figure 3C). Considering the concentration of IL-6 and CXCL-2, there was an increase of these mediators after 24 h of infection, and this increase was maintained after 72 h of infection compared to control animals (Figure 3D and F). In 5-LO^−/−^ mice, levels of TNF-α were lower 24 h after infection and higher after 72 h compared with cytokine levels of WT-infected mice (Figure 3A). Levels of IFN-γ were lower 24 h after infection in 5-LO^−/−^ mice and were not detectable after 72 h (Figure 3B). Levels of IL-1β, CXCL-1 and CXCL-2 were higher in 5-LO^−/−^ mice 24 h after infection compared with WT mice (Figure 3C, E and F). Levels of IL-6 and CXCL-1 were higher in BAL of 5-LO^−/−^ mice 72 h after infection compared with the same period in WT mice (Figure 3D and E). Overall, the cytokines and chemokines production in the lungs (Table 1) of 5-LO^−/−^ mice after infection was higher to that found in WT mice 72 h after infection. In addition to the cytokines mentioned above, we could also measure significant amounts of IL-10 in lungs (Table 1), but not in BAL (not detected, data not shown), of *P. brasiliensis*-infected mice. Levels of IL-10 were high after 24 h and dropped to background levels 72 h after infection in WT mice. Levels of IL-10 remained low and were below background levels in 5-LO^−/−^ mice (Table 1).

**Table 1:** Cytokines and chemokines concentrations in the lungs of WT and 5-LO^−/−^ mice.

Cytokine or chemokine	WT			5-LO^−/−^		
	NI	24 h	72 h	NI	24 h	72 h
TNF-α	18.76±37.52	1497±492.7*	445.2±504.0†	11.12±19.26	526.7±102.8‡	2243±518.6* † ‡
IFN-γ	249.7±139.5	858.5±76.02*	430.6±135.2†	306.9±103.6	279.6±142.9‡	621.4±192.8* † ‡
IL-1β	241.5±104.6	5868±422.0*	2223±1016* †	239.1±103.6	4708±930.5* ‡	5813±308.8* † ‡
IL-6	59.91±119.8	780.2±347.0*	28.07±47.35†	ND	1923±123.0* ‡	1045±664.9* † ‡
IL-10	188.7±51.95	559.0±43.64*	176.3±146.9†	157.9±84.52	30.61±37.54* ‡	57.55±40.83
CXCL1/KC	144.4±38.89	3721±1250*	774.1±470.5†	419.9±300.9	7184±959.2* ‡	2840±1352* † ‡
CXCL2/MIP-2	252.8±106.7	5970±1706*	650.0±562.0†	431.5±332.8	5676±428.9*	2916±1606* † ‡

Values represent the mean and standard deviation of cytokines and chemokines concentrations (pg/100 mg of tissue) in the lungs of WT and 5-LO^−/−^ mice. NI: not infected. ND: not detected.

* p<0.01; p<0.001 Statistical differences between groups of mice infected during the periods of time indicated (24 h or 72 h) compared with NI mice.

† p<0.05; p<0.01; p<0.001 Statistical differences between times after infection (24 h and 72 h) within the same group of mice (WT or 5-LO^−/−^).

‡ p<0.05; p<0.01; p<0.001 Statistical differences between 5-LO^−/−^ mice compared with WT mice in the time period indicated (24 h or 72 h) following infection.

### Pharmacological inhibition of 5-LO during infection with *P. brasiliensis* mimics the phenotype of 5-LO-deficient mice

**Figure 4:**
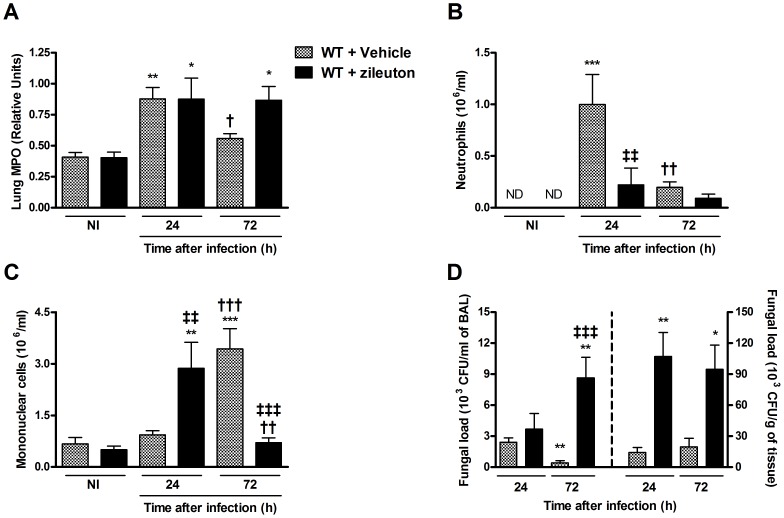
Effect of LT-synthesis blockade *in vivo* during infection with *P. brasiliensis*. WT mice were treated with zileuton (30 mg/Kg/day; 0.2 ml by gavage) before infection with *P. brasiliensis* yeasts. (A) MPO activity in the lungs of mice inoculated with *P. brasiliensis* yeasts was expressed in relative units. Control (NI) mice were injected with PBS and treated daily with the same volume of vehicle solution by the same route. BAL cells were obtained from mice 24 and 72 h after infection and neutrophils (B) and mononuclear cells (C) were counted and identified after staining. (D) Fungal burden was evaluated by counting the number of viable yeasts presents in BAL (left) and lung homogenates (right). The results were expressed as the mean ± SEM (n = 5). Symbol * represents significant difference (*p<0.05, **p<0.01, ***p<0.001) between infected mice compared to respective control group. Symbol ^†^ represents significant difference (^†^p<0.05, ^††^p<0.01, ^†††^p<0.001) to infected mice counterparts in evaluated periods. Symbol ^‡^ represents significant difference (^‡‡^p<0.01, ^‡‡‡^p<0.001) between WT and 5-LO^−/−^ mice in the respective periods evaluated. ND, not detected.

To determine the effects of pharmacological inhibition of 5-LO during *P. brasiliensis* infection, WT mice were treated daily with zileuton, a compound which inhibits the leukotrienes synthesis by complexing with an iron atom in the site of the 5-LO enzyme [34]. Overall, treatment with zileuton resulted in changes at inflammatory parameters which were remarkably similar to those of 5-LO^−/−^ mice (Figure 4). Indeed, there was greater MPO activity in the lungs (Figure 4A) but fewer neutrophil counts in BAL (Figure 4B), suggesting that neutrophils were trapped into the parenchyma and increased with time. Similarly, number of mononuclear cells early increased and were lower than vehicle-treated mice 72 h after infection (Figure 4C). Similar to that observed in 5-LO^−/−^ mice, zileuton-treated mice presented increased fungal burden in BAL and lung tissue after infection (Figure 4D).

### Absence or pharmacological inhibition of 5-LO decreased phagocytosis and killing of *P. brasiliensis* by macrophages

**Figure 5:**
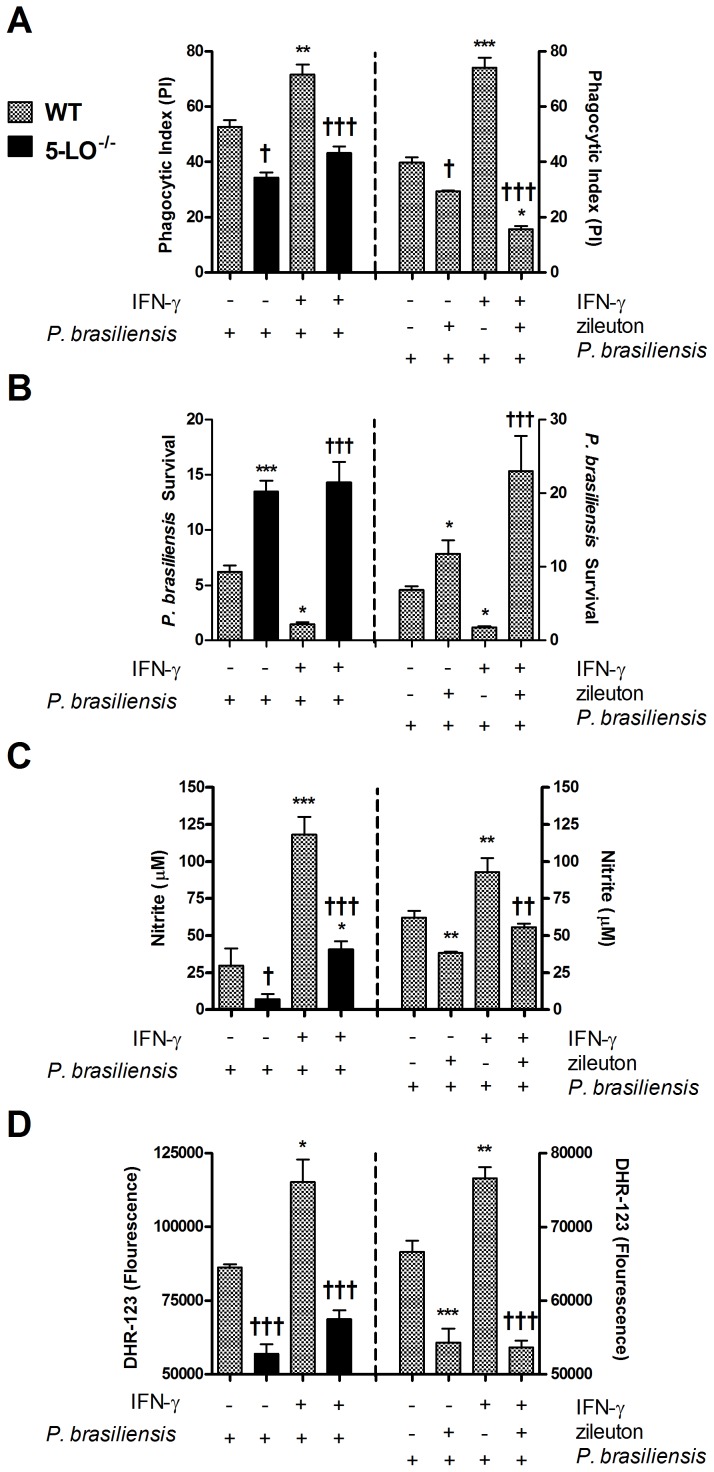
Phagocytic and fungicidal activity during infection with *P. brasiliensis in vitro*. (Left) Macrophages were harvested from the peritoneal cavity of WT and 5-LO^−/−^ mice and infected *in vitro* with *P. brasiliensis* yeasts. (Right) Peritoneal WT macrophages were treated with vehicle or zileuton (10 mM) for 30 min before infection *in vitro* with *P. brasiliensis* yeasts. Phagocytic index (A) and intracellular killing (B) were calculated 24 h after incubation. Killing assay was normalized according initial ingestion (PI) and presented as *P. brasiliensis* survival. The supernatants were harvested and nitrite concentrations (C) were measured. (D) ROS concentrations were determined by mean fluorescence intensity (DHR-123) in macrophage cultures after infection. Each value represents the mean ± SEM of triplicate cultures from two independent experiments. Symbol * represents significant difference (*p<0.05, **p<0.01, ***p<0.001) compared to WT infected group. Symbol ^†^ represents significant difference (^†^p<0.05, ^††^p<0.01, ^†††^p<0.001) between the same experimental group compared to different culture conditions.

Given the increased fungal burden observed in 5-LO^−/−^ or zileuton-treated mice, we examined the ability of macrophages from these mice to deal with the fungus *in vitro*. WT macrophages were capable of engulfing *P. brasiliensis* and this phagocytic activity was enhanced by IFN-γ (Figure 5A). Also, IFN-γ were able to significantly increase fungal killing (Figure 5B), which was associated with an increased NO (Figure 5C) and ROS production by infected macrophages (Figure 5D). In macrophages from 5-LO^−/−^ mice or treated with zileuton, *P. brasiliensis* phagocytosis was decreased, even in the presence of IFN-γ (Figure 5A). Moreover, decreased spontaneous phagocytosis or in response to IFN-γ was followed by increased fungal burden recovered from the macrophage cultures (Figure 5B) and lower NO and ROS production (Figure 5C and D). Essentially, similar results were also observed in alveolar macrophages from WT and 5-LO^−/−^ mice subjected to the same stimuli (Figure S1).

### LTB_4_ receptor antagonist decreased phagocytosis and fungicidal activity of macrophages infected with *P. brasiliensis*

**Figure 6:**
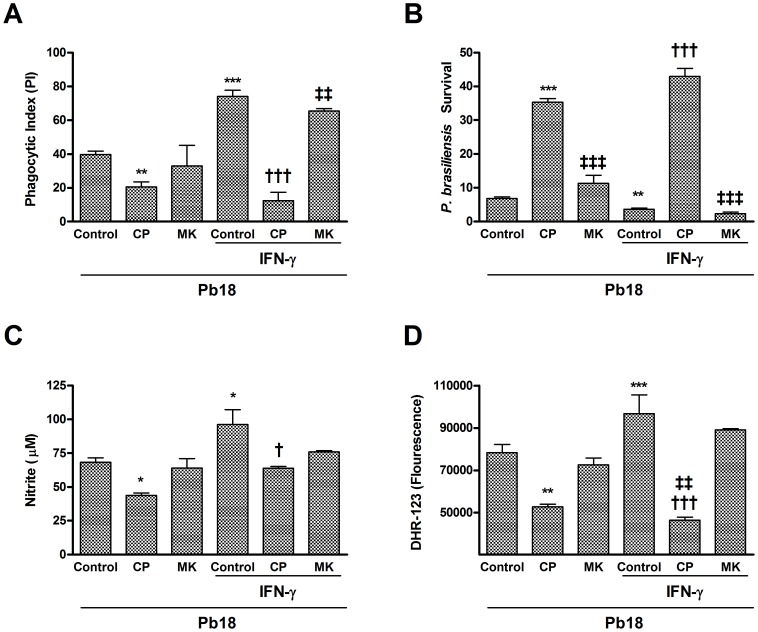
Effect of LTB_4_- or cysLT-receptor antagonists on macrophage phagocytosis and fungicidal activity against *P. brasiliensis*. Peritoneal WT macrophages were treated with vehicle, LTB_4_ receptor antagonist CP-105,696 (CP) (1 mM) or cysteinyl-LT receptor antagonist montelukast (MK) (10 mM) for 30 min before the addition of *P. brasiliensis* yeasts. Phagocytic index (A) and intracellular killing (B) were calculated 24 h after incubation. Killing assay was normalized according initial ingestion (PI) and presented as *P. brasiliensis* survival. The supernatants were harvested and nitrite concentrations (C) were measured. (D) ROS concentrations were determined by mean fluorescence intensity (DHR-123) in macrophage cultures after infection. Each value represents the mean ± SEM of triplicate cultures from two independent experiments. Symbol * represents significant difference (*p<0.05, **p<0.01, ***p<0.001) compared to control group (macrophages that were treated with vehicle and infected with *P. brasiliens* yeasts). Symbol ^†^ represents significant difference (^†^p<0.05, ^†††^p<0.001) compared to IFN-γ control group (macrophages that were activated with IFN-γ, treated with vehicle and infected with *P. brasiliens* yeasts). Symbol ^‡^ represents significant difference (^‡‡^p<0.01) between the same experimental group compared to different culture conditions.

Our previous experiments established a role for 5-LO pathway products but did not identify the metabolite responsible for the endogenous antifungal activity. Therefore, we used a strategy involving selective receptor antagonists (LTB_4_ receptor antagonist - CP-105,696 or cysteinyl-LT receptor antagonist - montelukast) to determine the individual contributions of LTB_4_ and cysLTs, specific 5-LO metabolites, in phagocytosis and fungicidal activity. As shown in Figure 6, treatment of macrophages with the LTB_4_ receptor antagonist, but not the cysteinyl-LT receptor antagonist, decreased phagocytosis (either spontaneous or enhanced by IFN-γ) and *P. brasiliensis* killing (Figure 6A and B). Also, there was reduced production of NO and ROS after treatment of macrophages with LTB_4_ receptor antagonist, fact that was not observed in macrophages treated with the cysteinyl-LT receptor antagonist (Figure 6C and D).

### *P. brasiliensis* infection induces LTB_4_ synthesis

**Figure 7:**
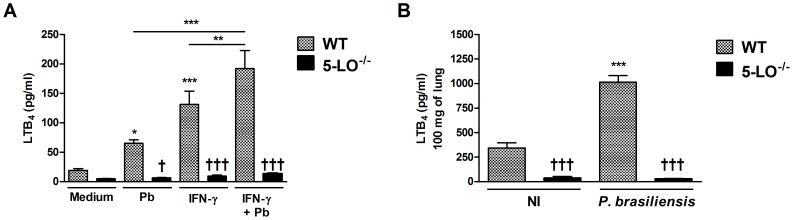
LTB_4_ production after fungal infection. (A) Enzyme immunoassay quantification of LTB_4_ concentrations in alveolar macrophages cultures after 24 h of incubation with *P. brasiliensis* yeasts. Data are presented as mean ± SEM and are representative of one experiment (n = 8). Symbol * represents significant difference (*p<0.05, **p<0.01, ***p<0.001) when compared to uninfected cultures (only medium) or between indicated groups. Symbol ^†^ represents significant difference (^†††^p<0.001) between WT and 5-LO^−/−^ macrophages under the conditions mentioned. (B) Lung LTB_4_ concentration in WT and 5-LO^−/−^ mice. Quantification of LTB_4_ in lungs from uninfected- (control - NI) or infected mice with *P. brasiliensis* evaluated 24 h after inoculation. Leukotriene concentrations in supernatant of homogenized lungs (100 mg) were expressed as pg/ml. Data were expressed as the mean ± SEM and are representative of one experiment (n = 6). Symbol * represents significant difference (***p<0.001) when compared to uninfected mice. Symbol ^†^ represents significant difference (^†††^p<0.001) between WT and 5-LO^−/−^ mice under the conditions mentioned.

Having established the importance of endogenously produced LTB_4_ for phagocytic capacity of macrophages at levels similar to those observed after stimulation with IFN-γ, we next investigated if *P. brasiliensis* phagocytosis would result in the LTB_4_ synthesis, in the presence or not of IFN-γ. As indicated in Figure 7A, control cultures (only medium) had very low LTB_4_ levels, and these were similar in WT and 5-LO^−/−^ macrophages. However, WT-infected macrophages showed increased LTB_4_ production 24 h after *P. brasiliensis*-macrophages interaction and this level was significantly enhanced in presence of IFN-γ stimulation alone or after stimulation and interaction with *P. brasiliensis*. Macrophage production of LTB_4_ for all conditions evaluated was markedly reduced in 5-LO-deficient macrophages.

Further, we measured LTB_4_ production in the lungs of infected mice (Figure 7B). WT and 5-LO^−/−^ mice were infected with *P. brasiliensis* yeast cells and the production of this lipid mediator was determined 24 h after infection. Uninfected WT mice displayed low levels of LTB_4_ whereas infection with *P. brasiliensis* induced increased lung LTB_4_ production. LTB_4_ levels were significantly lower in the lungs of 5-LO-deficient mice.

### Addition of LTB_4_ restores phagocytosis and fungicidal activity of macrophages

**Figure 8:**
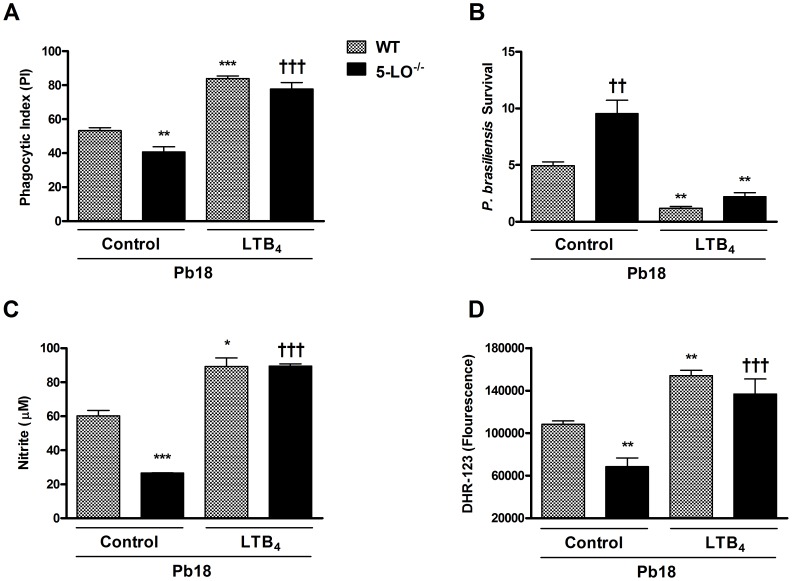
Effects of exogenous LTB_4_ on phagocytosis and fungicidal activity *in vitro* during infection with *P. brasiliensis*. Peritoneal WT and 5-LO^−/−^ macrophages were treated with LTB_4_ (100 mM) for 30 min before the addition of *P. brasiliensis* yeasts. Phagocytic index (A) and intracellular killing (B) were calculated 24 h after incubation. Killing assay was normalized according initial ingestion (PI) and presented as *P. brasiliensis* survival. The supernatants were harvested and nitrite concentrations (C) were measured. (D) ROS concentrations were determined by mean fluorescence intensity (DHR-123) in macrophage cultures after infection. Each value represents the mean ± SEM of triplicate cultures from two independent experiments. Symbol * represents significant difference (*p<0.05, **p<0.01, ***p<0.001) compared to WT infected group. Symbol ^†^ represents significant difference (^††^p<0.01, ^†††^p<0.001) between the same experimental group compared to different culture conditions.

Previous experiments suggested that the LTB_4_ receptor was the major signaling pathway that accounted for the action of 5-LO derived products in the context of *P. brasiliensis* infection. We therefore evaluated whether addition of LTB_4_ could induce phagocytosis and fungicidal activity directly and overcome the phenotype of 5-LO-deficient mice. As shown in Figure 8, pretreatment of WT macrophages with LTB_4_ enhanced the phagocytic activity, *P. brasiliensis* killing and NO and ROS production. More importantly, addition of LTB_4_ reversed the phenotype observed in 5-LO^−/−^ macrophages, causing greater phagocytosis, fungal killing and NO and ROS production (Figure 8). The same results were observed with alveolar macrophages (Figure S1).

### LTB_4_ increases nitric oxide-dependent killing of *P. brasiliensis*

**Figure 9:**
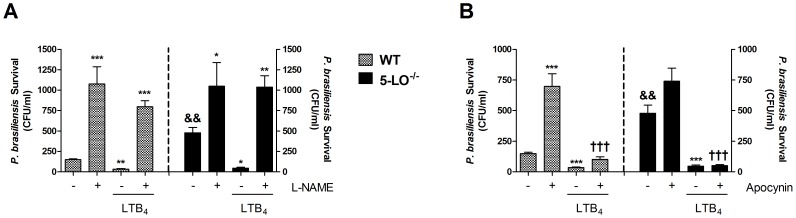
Role of NO and ROS in the fungal killing induced by LTB_4_ in macrophages. Alveolar macrophages were pretreated with the iNOS inhibitor L-NAME (1 mM) (A) or the NADPH oxidase inhibitor apocynin (1 mM) (B) for 30 minutes before the addition of *P. brasiliensis* yeasts. Cultures were added with or without LTB_4_ (100 mM) 30 minutes later. Fungicidal assay was performed and fungal survival was represented as number of colony forming units (CFU) per ml of culture. Data are expressed as the mean ± SEM of triplicate cultures from one experiment. Symbol * represents significant difference (*p<0.05, **p<0.01, ***p<0.001) compared to untreated macrophages. Symbol ^†^ represents significant difference (^†††^p<0.001) compared to macrophages culture treated with the respective inhibitor. Symbol ^&^ represents significant difference (^&&^p<0.01) compared with the same culture condition in WT macrophages.

As the results described above indicated that decreased NO and ROS generation was associated with a reduction in the ability of macrophages to kill *P. brasiliensis* yeasts, we evaluated the effect of LTB_4_ in modulating macrophage antifungal activity by these reactive species. For these experiments, macrophages were pretreated with L-NAME (1 mM), a NO synthase inhibitor or apocynin (1 mM), a NADPH oxidase inhibitor, and then stimulated with LTB_4_. As shown in Figure 9A and B, inhibition of NO or ROS production abolished the ability of WT- or 5-LO^−/−^ macrophages to control *P. brasiliensis* infection, increasing the number of viable yeasts. In the presence of L-NAME, exogenous LTB_4_ failed to overcome this defect (Figure 9A). However, inhibition of ROS by apocynin increased *P. brasiliensis* survival in macrophages and this phenotype was reversed by treatment with LTB_4_ (Figure 9B). Taken together, these data suggest that NO generated by iNOS activity plays an important role in the enhanced killing induced by LTB_4_ in macrophages.

## Discussion

The present study demonstrates the effects and mechanisms by which 5-LO and its metabolic products positively participate in host defense against the pathogenic fungus *P. brasiliensis*. Infection with *P. brasiliensis* was uniformly fatal in 5-LO-deficient mice and the mechanisms accounting for this phenotype were associated with exacerbated lung pathology and higher pulmonary fungal load. The susceptibility of 5-LO^−/−^ mice was also correlated with lower macrophage activation, resulting in reduced phagocytosis and NO dependent-killing of *P. brasiliensis* yeasts. Exogenous LTB_4_ restored these functions in 5-LO-deficient macrophages. Previous studies have shown that in a model of *Histoplasma capsulatum* infection, another important pathogenic dimorphic fungus, reductions in leukotriene synthesis caused by MK 886 administration, a 5-LO pathway inhibitor [35], or in 5-LO-deficient mice [36], resulted in significant mortality in mice and increased lung CFU at 7 and 14 days post infection. These results are consistent with a pivotal role of 5-LO metabolites in the protective host response seen in this model of *P. brasiliensis* infection.

Leukotrienes are chemotactic for neutrophils [37] and T lymphocytes [38] and can also contribute to leukocyte recruitment indirectly by augmenting cytokines and chemokines production [39]. Thus, we assessed whether the increased susceptibility of 5-LO^−/−^ mice was associated with the leukocyte recruitment profile to the site of infection. While in WT mice neutrophil influx into pulmonary parenchyma peaked at 24 h after infection, in 5-LO^−/−^ mice this influx was continued, resulting in exacerbated lung inflammation and increased pulmonary tissue damage. Previous studies have demonstrated that neutrophil recruitment into the lungs occurs in a sequence of events involving their sequestration in pulmonary vessels, migration from blood to interstitium and then migration from the interstitium to alveolar spaces across epithelial cells. Cytokines (TNF-α, IL-1β, and IL-6), chemokines (CXCL-1 and CXCL-2) and lipid mediators such LTB_4_ and platelet-activating factor (PAF), triggered by activation of resident cells in the inflamed area, regulate this migration in concert with cell adhesion molecules [40]. It is clear that different mechanisms account for the accumulation of neutrophils in the interstitium and in airspaces in models of pulmonary inflammation [41]. Therefore, one can envisage the situation observed in the current study in which neutrophil accumulation in airspace is decreased, but not in lung tissue. These results demonstrate clearly that 5-LO-derived molecules, likely LTB_4_, are crucial for transepithelial migration but not to accumulation in the lung parenchyma.

Neutrophil and macrophage recruitment into the pulmonary parenchyma was not sufficient to control *P. brasiliensis*, as 5-LO^−/−^ mice had greater number of viable yeasts recovered in the lungs. It is interesting that, despite the high number of macrophages in lung tissues of 5-LO^−/−^ mice 24 h after infection, these cells seemed to be less activated. Hence, macrophages of 5-LO^−/−^ mice did not up-regulate MHC-II expression upon *P. brasiliensis* infection as their WT counterparts. Macrophages are believed to be important in the initial containment of the microorganisms through nonspecific or natural immune mechanisms, and the expression of MHC-II in macrophages is indicative of a preserved and active macrophage function in patients with PCM, in addition to its essential function in antigen presentation [42], [43]. Therefore, these data suggest that 5-LO-derived metabolites are essential for macrophage activation and function during interaction with *P. brasiliensis* yeasts.

Cytokine production during acute infection in murine PCM is extremely important because it influences the fate of immune response, which may lead to susceptibility or resistance [44]–[50]. Experimental studies and patients with PCM indicate that resistance to infection is dependent on the activity of T helper cells and macrophages/monocytes mediated by IFN-γ and TNF-α. The synergistic effect between these two cytokines is essential for the activation of the host cells and efficiently fungicidal activity against *P. brasiliensis* [51], [52]. In accordance, our results suggested that after 24 h of infection, there is lower TNF-α and IFN-γ production in alveolar space of 5-LO^−/−^ mice. These decreased cytokines production may have contributed to increased susceptibility to *P. brasiliensis* infection, associated with minor cell activation (lower expression of MHC-II in 5-LO^−/−^ macrophages) and failure in fungicidal activity. These latter defects in combination would result in elevated pulmonary fungal burden. It was interesting to note that there was a delayed production of TNF-α in 5-LO^−/−^ mice. If lack of early response may contribute to inability to deal with infection, late production may have contributed to the enhanced inflammatory response and tissue injury. Although TNF-α is predominantly produced by activated mononuclear phagocytes *in vivo*, a number of other resident and inflammatory cells, including endothelial cells, mast cells, smooth muscle cells, B cells, and T cells, can secrete TNF-α [53]. Based on our findings, it appears that if the initial response by macrophages fails, other pulmonary cells may release this and potentially other cytokines in response to high fungal burden in the lungs of 5-LO^−/−^ mice.

Conversely, we observed an important increase of cytokines production, TNF-α, IFN-γ, IL-1β, IL-6 and CXCL-1/2 in lung tissue of 5-LO^−/−^ mice. Therefore, the higher amounts of these cytokines and chemokines, together with the neutrophil accumulation in the lungs of 5-LO-deficient mice, might contribute to exacerbation of inflammatory response leading to tissue damage and loss of lung functions. IL-10 is considered the most important anti-inflammatory cytokine since it may reduce the production of inflammatory mediators and suppress effector functions of macrophages and other immune cells. Given that our results showed reduced concentration of IL-10 in the lungs homogenates of 5-LO^−/−^ mice during infection with *P. brasiliensis*, this may have contributed to the exacerbated production of pro-inflammatory cytokines and chemokines observed in these animals.

Lipoxins, such as lipoxin A_4_ (LXA_4_), are 5-LO-derived products which decrease macrophage function and which have known anti-inflammatory activity *in vivo*. In the absence of 5-LO, there could also be decreased LXA_4_ production and consequent facilitation of inflammation. This molecule was not investigated here as we focused on the leukotriene pathway and we, therefore, cannot discard the involvement of LXA_4_ in our system. Specific blockers of LXA_4_ production or action are not widely available, making it difficult to examine the exact role of this molecule *in vivo*. Future studies should define the role of LXA_4_ as specific tools become available.

In this study, we used both genetic and pharmacologic approaches to demonstrate that endogenously produced LTB_4_ promoted macrophage phagocytosis and *P. brasiliensis* killing. We observed that both macrophages elicited from the peritoneum or derived from the lungs of 5-LO^−/−^ mice exhibited reduced phagocytosis and fungal killing. The same phenotype was found when peritoneal macrophages were pretreated with zileuton, a drug that block 5-LO activity. It is likely that LTB_4_ is the endogenous 5-LO product responsible for this effect, since LTB_4_ receptor antagonist, but not cysteinyl-LT receptor one, also reduced macrophage activities. Moreover, exogenous LTB_4_ was capable of restoring antifungal functions of macrophages to levels similar to observed in WT macrophages. Our results are consistent with published data showing the capacity of LTB_4_ to induce polymorphonuclear neutrophils and macrophages phagocytosis [44]–[46]. Recently, it was demonstrated that LTB_4_ and its high affinity receptor BLT1 signaling are central determinants of dectin-1 expression and of host responses to fungi [54]. Dectin-1, a C-type lectin, is the major receptor on macrophages for β-1,3-glucan, a polymer of glucose present in the fungal cell wall that stimulates phagocytosis, production of inflammatory cytokines and reactive species [55]. Dectin-1 engagement also activates phospholipase A2 with subsequent production of eicosanoid lipid mediators including cyclooxygenase-derived prostanoids and 5-LO-derived leukotrienes such as LTB_4_ [56], [57]. Previous studies highlighted the participation of dectin-1 in *P. brasiliensis* recognition, internalization and consequent activation of the immune response against the fungus [58]. Future research should detail whether the expression of this receptor is controlled by 5-LO and its metabolites in macrophages and *in vivo*.

The initial contact of *P. brasiliensis* to the host is handled by phagocytic cells, which constitute an important part of innate defense. The fungus is internalized by macrophages *in vivo* and *in vitro*, and multiplies in monocytes and macrophages that are not activated. Only monocytes and macrophages activated with IFN-γ and TNF-α have fungicidal activity against *P. brasiliensis* [59]. The results presented here demonstrated that *P. brasiliensis*-macrophage interaction induced high LTB_4_ production. Interestingly, we verified that IFN-γ-primed macrophages enhanced LTB_4_ levels. Also, it appears that IFN-γ and *P. brasiliensis* had a synergistic effect on macrophages since the two stimuli together enhanced LTB_4_ concentrations in cultures. Our study demonstrates for the first time that IFN-γ induces and facilitates production of LTB_4_ by macrophages during *P. brasiliensis* infection *in vitro*. Therefore, our work contributes substantially to the knowledge of the literature, indicating that the participation of IFN-γ in the control mechanisms of infection by macrophages was partly promoted by the production of LTB_4_.

One of the characteristics of activated macrophages is the increase in nitric oxide (NO) production. NO, synthesized by induced NO synthase (iNOS), possesses known antimicrobial activity against *P. brasiliensis* [60]. Through reaction of NO with superoxide anion (O_2_^−^), peroxynitrite (ONOO^−^) is produced, which is cytotoxic and important for the cellular immunity. Macrophages also produce hydrogen peroxide (H_2_O_2_), which is an important fungicidal agent [61]. Previous studies have shown that leukotrienes enhance reactive species production in macrophages infected with many microorganisms [19], [36], [44], [62]. A significant increase in NO and ROS production was observed in WT macrophage cultures incubated with *P. brasiliensis* after activation with IFN-γ. There was decreased levels of NO and ROS in infected 5-LO-deficient macrophages. This phenotype observed in 5-LO^−/−^ macrophages was reversed by LTB_4_. We also observed increased NO and ROS production in WT macrophages cultures after LTB_4_ addition at levels similar to macrophages activated with IFN-γ. Therefore, our data show that activation of 5-LO contributed to enhanced macrophage activity by facilitating the generation of NO and ROS. Interestingly, we showed that clearance of *P. brasiliensis* is dependent on NO production only, as macrophages treated with apocynin (NADPH oxidase inhibitor) but not with L-NAME (iNOS inhibitor) reversed the killing after LTB_4_ addition in cultures. Therefore, our data suggest that NO generated by iNOS activity plays an important role for the enhanced antifungal activity induced by LTB_4_ in macrophages. ROS production is also dependent on 5-LO-induced LTB_4_ but is not important for killing of the fungus.

Recently, two studies have been published investigating the involvement of the LT pathway in *P. brasiliensis* infection. These studies differ with respect to the role of these mediators during infection. The first one showed that levels of leukotrienes and lipoxins were increased in WT-infected mice and that 5-LO^−/−^ mice exhibited diminished fungal growth and increased survival rate compared to WT mice. These data indicate that 5-LO derived products might have a critical role in the exacerbation and severity of PCM [28]. Another study investigated the involvement of leukotrienes in the early stages of experimental PCM in selected mouse lines endowed with maximal or minimal acute inflammatory reactivity, and designated AIRmax and AIRmin, respectively [29]. Mice treated with the 5-LO inhibitor (MK 886) presented increased fungal recovery, showing that endogenous production of leukotrienes is associated with a protective immune response. Our study differs in several aspects to the first one [28], especially regarding the route of infection. Our murine experimental model used the intratracheal route that better mimics the infection in the human host. This experimental design allowed us to evaluate the acute inflammatory response triggered against the fungus while the model described in the previous study, developed from the intravenous infection, allowed to investigate chronic/systemic aspects of *P. brasiliensis* infection. Therefore, it appears that innate immune response differs depending on the initial organ infected by the microorganism and the route of inoculation is important to determine this response in host. Also, our results reinforce the data presented in the second study [29] since we found that leukotrienes, especially LTB_4_, are involved in the mechanisms that control fungal infection. We verified that 5-LO products play an important role in inflammatory cell recruitment and cytokines production. Importantly, LTB_4_ released in the lungs could activate resident cells and enhance macrophages function, like phagocytosis and NO-dependent fungal killing, contributing to resistance of host against *P. brasiliensis* infection. In addition, our study provides important mechanistic insights into the participation of 5-LO-derived products during *P. brasiliensis* infection.

In summary, we have shown that the absence of 5-LO resulted in death of the host infected with *P. brasiliensis*. Increased mortality was associated with changes in the recruitment of inflammatory cells in the early stages of infection, fungal proliferation and exacerbated lung inflammation. Absence or blockade of 5-LO decreased phagocytosis and NO-dependent fungicidal activity of macrophages. Furthermore, it is suggested that LTs, especially LTB_4_, are the major 5-LO-dependent mediator of *P. brasiliensis* phagocytosis and killing. These findings indicate that an intact 5-LO system is required for efficient pulmonary antifungal host defense against *P. brasiliensis* infection.

## Supporting Information

Figure S1 **Phagocytic and fungicidal activity during infection with** ***P. brasiliensis in vitro*** **.** Alveolar macrophages were harvested from lung lavage of WT and 5-LO^−/−^ mice and infected *in vitro* with *P. brasiliensis* yeasts. Phagocytic index (A) and intracellular killing (B) were calculated 24 h after incubation. Survival was calculated considering the remaining colony forming units (CFU) from the killing assay in function of the phagocytic index (PI). The supernatants were harvested and nitrite concentrations (C) were measured. (D) ROS concentrations were determined by mean fluorescence intensity (DHR-123) in macrophage cultures after infection. Each value represents the mean ± SEM of triplicate cultures from one experiment. Symbol * represents significant difference (**p<0.01, ***p<0.001) compared to WT infected group. Symbol ^†^ represents significant difference (^†^p<0.05, ^††^p<0.01, ^†††^p<0.001) between WT and 5-LO^−/−^ macrophages at the same culture conditions.
(TIF)
