# Supplementary figures and images for: The Pivotal Role of 5-Lipoxygenase-Derived LTB4 in Controlling Pulmonary Paracoccidioidomycosis
Source: PLoS Negl Trop Dis. 2013 Aug 22;7(8):e2390. doi: 10.1371/journal.pntd.0002390 (PMC3749973; doi:10.1371/journal.pntd.0002390)

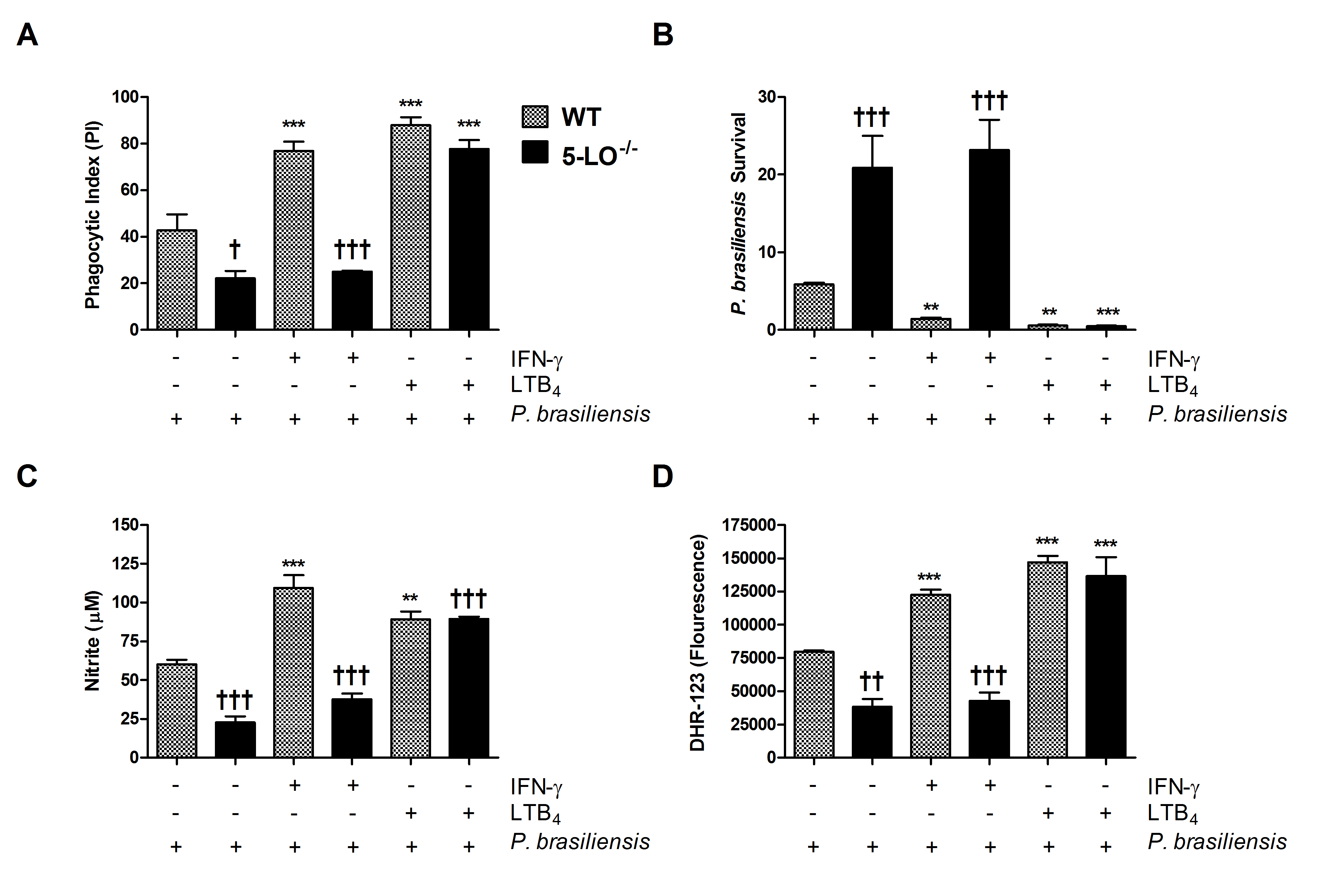

Supplement: Figure S1 — Phagocytic and fungicidal activity during infection with P. brasiliensis in vitro . Alveolar macrophages were harvested from lung lavage of WT and 5-LO−/− mice and infected in vitro with P. brasiliensis yeasts. Phagocytic index (A) and intracellular killing (B) were calculated 24 h after incubation. Survival was calculated considering the remaining colony forming units (CFU) from the killing assay in function of the phagocytic index (PI). The supernatants were harvested and nitrite concentrations (C) were measured. (D) ROS concentrations were determined by mean fluorescence intensity (DHR-123) in macrophage cultures after infection. Each value represents the mean ± SEM of triplicate cultures from one experiment. Symbol * represents significant difference (**p<0.01, ***p<0.001) compared to WT infected group. Symbol † represents significant difference (†p<0.05, ††p<0.01, †††p<0.001) between WT and 5-LO−/− macrophages at the same culture conditions. (TIF) [file pntd.0002390.s001.tif]
